# Blue 405 nm LED light effectively inactivates bacterial pathogens on substrates and packaging materials used in food processing

**DOI:** 10.1038/s41598-023-42347-z

**Published:** 2023-09-19

**Authors:** Hanyu Chen, Yifan Cheng, Carmen I. Moraru

**Affiliations:** 1 Department of Food Science, Cornell University, Ithaca, NY 14853 USA; 2 Department of Food Science, Virginia Polytechnic Institute and State University, Blacksburg, VA 24061 USA

**Keywords:** Food microbiology, Applied microbiology

## Abstract

This study investigates the antimicrobial effectiveness of 405 nm light emitting diodes (LEDs) against pathogenic *Escherichia coli* O157:H7, *Listeria monocytogenes*, *Pseudomonas aeruginosa*, *Salmonella* Typhimurium, and *Staphylococcus aureus*, in thin liquid films (TLF) and on solid surfaces. Stainless steel (SS), high density polyethylene (HDPE), low density polyethylene (LDPE), and borosilicate glass were used as materials typically encountered in food processing, food service, and clinical environments. Anodic aluminum oxide (AAO) coupons with nanoscale topography were used, to evaluate the effect of topography on inactivation. The impact of surface roughness, hydrophobicity, and reflectivity on inactivation was assessed. A 48 h exposure to 405 nm led to reductions ranging from 1.3 (*E. coli*) to 5.7 (*S. aureus*) log CFU in TLF and 3.1 to 6.3 log CFU on different solid contact surfaces and packaging materials. All inactivation curves were nonlinear and followed Weibull kinetics, with better inactivation predictions on surfaces (0.89 ≤ *R*^2^ ≤ 1.0) compared to TLF (0.76 ≤ *R*^2^ ≤ 0.99). The fastest inactivation rate was observed on small nanopore AAO coupons inoculated with *L. monocytogenes* and *S. aureus*, indicating inactivation enhancing potential of these surfaces. These results demonstrate significant promise of 405 nm LEDs for antimicrobial applications in food processing and handling and the healthcare industry.

## Introduction

Despite significant public and private investments to mitigate transmission of bacterial pathogens in the food sector, bacterial pathogens transmitted via certain foods, particularly raw foods, as well as water and various environmental sources remain a major cause of illness in both developed and developing countries, with *Escherichia coli* O157:H7, *Listeria monocytogenes*, *Pseudomonas aeruginosa*, *Salmonella*, and *Staphylococcus aureus* being some of the main culprits^1,2^. These pathogens are also of concern in clinical environments and other areas of human activity. Methods commonly used for inactivating these pathogenic microorganisms in foods include thermal treatments and the use of antimicrobials^3^. For decontaminating food-contact surfaces, packaging materials, utensils or other types of surfaces, sanitizers are often applied^4^. While these treatments are effective, their usefulness has been often times shadowed by undesired effects on food quality or the environment^5,6^. Additionally, some were shown to lead to increased antimicrobial resistance in microorganisms^7–9^. Considerable efforts have been made in recent years to develop novel decontamination approaches to combat foodborne illness^10,11^ and nosocomial infections^12,13^, which can also avoid the limitations associated with conventional antimicrobial methods. These include novel antibiotics^5^ and antimicrobial treatments that target simultaneously multiple metabolic processes key to microbial survival^14,15^.

Light based treatments, including continuous ultraviolet light (UV) and pulsed light (PL), both inducing DNA damage primarily as a result of absorption of wavelengths in the UV range by the bacterial DNA, have demonstrated microbicidal effects^16,17,18^. However, limitations such as the low efficiency of the light sources, particularly at refrigeration temperatures^19^, a lack of systems that offer three-dimensional exposure of complex and large objects^20^, as well as the detrimental effects of direct UV exposure to mammalian cells, restrict the use of these technologies^17,21^. Light emitting diodes (LEDs) emitting electromagnetic radiation in the visible wavelengths have garnered increasing attention in recent years as a safe^22,23^, energy efficient^24,25^, non-UV-based microbial decontamination technology in the food industry^26^ and clinical environments^27,28^. LED lighting is also increasingly used in horticulture, to increase plant photosynthesis and phototropism, or to extend the shelf life of fresh produce in the postharvest stage^24^. For example, colored LED lights were reported to delay the ripening in tomatoes and broccoli through retarding the chlorophyll degradation and slowing down sugar loss, thus extending their shelflife^29–31^. Exposure to blue LED light of 456 nm successfully reduced fungal colonization of *Penicillium digitatum* on the surface of tangerine fruits^32^. Furthermore, Xu et al.^33^ found that blue LEDs can increase the antioxidant activity and antioxidant enzyme activity in strawberries. In addition, an increase in vitamin C content was observed in cabbage treated by blue LEDs^34^.

Blue light with wavelengths from 405 to 470 nm has been reported to achieve greater inactivation compared to other regions of visible light^7^. Light in this wavelength range stimulates endogenous microbial porphyrin molecules to produce oxidizing reactive oxygen species (ROS), predominantly singlet oxygen, which may attack cellular DNA, lipids, and proteins, leading to cell death^28,35^. ROS may preferentially oxidize DNA in the cell membrane, and cause DNA damage by targeting the guanine bases, and formation of the oxidized derivative 8-hydroxy-deoxyguanosine (8-OHdG)^36,37^. Previous work demonstrated that up to 90% of *Staphylococcus aureus* cells can be photodynamically inactivated using 400 to 420 nm visible light, with maximum inactivation achieved at 405 nm^38^. A significant advantage of 405 nm LEDs is that that they can be used for a continuous, long-term (e.g., hours to days), ‘background’ treatment that can effectively control microbial contamination in food processing or medical environments, without interfering with normal human activities, since exposure to this treatment is not harmful to humans.

Blue LEDs are effective against both gram-negative and gram-positive bacteria, with a general trend showing gram-positives being more susceptible than gram-negatives^39,40^. Exposure of gram-positive *L. monocytogenes* suspended in a liquid to a blue light dose of 108 J/cm^2^ resulted in 5-log_10_ reduction, while the gram-negative *E. coli* reached a similar reduction at a significantly higher light dose, of 288 J/cm^40^. Since the existing information is limited, it is important to conduct challenge studies on a wide variety of Gram-positive and Gram-negative bacteria, to obtain an accurate assessment of the microbial inactivation effectiveness of blue LEDs.

There are several common contamination scenarios in the food and the health care industry that could benefit from LED treatment. One involves wet conditions, since bacteria can reside in static droplets and/or thin layers of liquid on the surface of food, packaging materials, utensils, or equipment. We recently demonstrated that the presence of thin liquid films (TLFs) and droplets on solid surfaces can drastically alter the spatial distribution of bacterial cells, diminishing penetration of UV light into the bacterial suspension and substantially diminishing inactivation^41^. This issue needs to be investigated for blue LED light exposure as well. On the other hand, certain surface modifications can maximize the repulsion between bacterial cells and abiotic surfaces^42,43^. Recent studies by our group have shown that anodic alumina surfaces with nanoscale cylindrical pores with diameters smaller than 25 nm can reduce attachment of both Gram-positive and Gram-negative foodborne pathogens^16,17^. This could also have positive implications on the antibacterial effectiveness of light-based treatments on such surfaces, which are worth exploring.

Therefore, the objective of this study is to investigate the effectiveness of 405 nm LEDs on several foodborne pathogens, both in liquid suspensions (TLFs) and on the surface of packaging and other materials commonly used in food or medical environments, including on surfaces with nanoscale topography. Both gram-positive bacteria (*L. monocytogenes* and *S. aureus*) and gram-negative bacteria (*E. coli*, *Salmonella* Typhimurium, *Pseudomonas aeruginosa*) of relevance for the food industry and clinical environments were used as challenge microorganisms. *S. aureus* is an opportunistic pathogen that can cause both food poisoning as well as invasive and potentially life-threatening infections^2, 20^. *E. coli*, *Salmonella*, and the biofilm former *Pseudomonas* spp. cause diarrheal and chronic infections^44^, while *L. monocytogenes* is feared due to its ability to grow at low temperatures and its potential to cause severe illness, especially to immunocompromised individuals^45^. Previous studies have shown that the effectiveness of UV light treatments is negatively impacted by low temperatures such as refrigeration conditions^46,47^. Therefore this study was conducted at refrigeration temperatures to examine the effectiveness of blue LED treatments in such conditions, and probe the possibility of using 405 nm blue LED as a disinfection treatment at low temperatures.

## Results and discussion

### Inactivation of bacteria in thin liquid films

**Figure 1:**
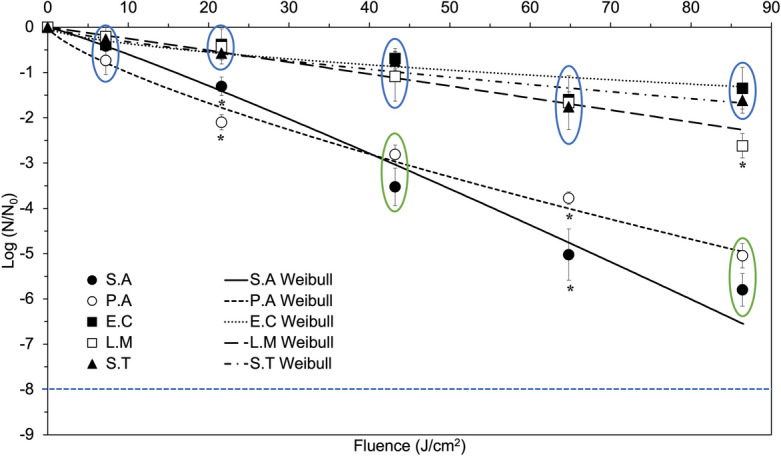
Inactivation of *E. coli, L. monocytogenes, S.* Typhimurium*, P. aeruginosa, S. aureus* in 1.2-mm thick liquid suspension by exposure to continuous 405 nm LEDs of an irradiance of approximately 0.5 mW/cm^2^. Data points represent means and error bars represent one standard deviation (n = 3). The blue dotted line denotes the limit of detection (LOD). Asterisks denote data points that are significantly different from the others at the same 405 nm LED exposure doses (*p* < 0.05), whereas circles denote clusters of data points that are not significantly different within the circle (*p* > 0.05). Circles of different colors indicate data points are significantly different between two circles at the same 405 nm LED exposure doses (*p* < 0.05).

Figure 1 shows the survivor ratios for the 405 nm LED treatments of liquid bacterial suspensions of *E. coli*, *L. monocytogenes*, *S.* Typhimurium, *S. aureus* and *P. aeruginosa* with an initial population density of 10^9^ CFU/mL and a suspension thickness of 1.2 mm, exposed to at an irradiance of 0.5 mW/cm^2^ for up to 48 h (86.4 J/cm^2^). The data shows that the counts of all tested pathogens decreased nonlinearly with treatment time, but there was a large variability in bacterial susceptibility to 405 nm treatment among the treated strains. The inactivation levels shown in Fig. 1 are about half of the inactivation levels reported in earlier studies for aqueous suspensions of bacteria of similar thickness, under constant stirring^39,40^. This difference may be caused by the higher accessibility of the 405 nm light exposure to bacteria due to the constant stirring and the lower bacterial cell concentration used in the previous studies compared to the current study. At the highest cumulative dose of 86.4 J/cm^2^ (48 h exposure), two susceptibility clusters to 405 nm treatment were identified based on the final inactivation levels reached. *E. coli* (1.3 ± 0.46 log reduction), *S.* Typhimurium (1.6 ± 0.28 log reduction), *L. monocytogenes* (2.6 ± 0.27 log reduction) were in the more resistant cluster, while *P. aeruginosa* (5.0 ± 0.27 log reduction) and *S. aureus* (5.8 ± 0.36 log reduction) were in the more susceptible cluster. No correlation between susceptibility to 405 nm light and cell wall structure, as indicated by gram status, was observed. The reduction for all five strains was similar (*p* > 0.05) for a cumulative dose of less than 7.2 J/cm^2^ (4 h exposure); the susceptibility differences between the two clusters became significant (*p* < 0.05) above a cumulative fluence of 21.6 J/cm^2^ (12 h exposure). In the lower susceptibility cluster, no significant difference in inactivation kinetics were observed among *E. coli*, *L. monocytogenes*, and *S.* Typhimurium for cumulative dose of 64.8 J/cm^2^ (36 h) or less. However, in the last 12 h of treatment, *L. monocytogenes* showed faster inactivation rates, and eventually reached a significantly higher log reduction than *E. coli* and *S.* Typhimurium at the highest fluence level (86.4 J/cm^2^). In the higher susceptibility cluster, the prolific biofilm formers *P. aeruginosa* and *S. aureus* appeared to be less resistant to 405 nm. No significant difference in final inactivation levels was observed between *S. aureus* and *P. aeruginosa* (*p* > 0.05), and no inactivation plateau was observed for these two strains.

All inactivation curves were fitted to the Weibull model, and the kinetic model parameters are shown in Table 1. The experimental data for all strains except *E. coli* showed a good fit with the Weibull model, with 0.94 ≤ *R*^2^ ≤ 0.99. The fit for *E. coli* was weaker, with an *R*^2^ = 0.77, which was likely due to the large variability of the data for this highly resistant strain. Overall, the inactivation results suggested that continuous 405 nm LED exposure for up to 48 h can effectively inactivate various foodborne pathogens. Since 405 nm blue light has been shown not to have any long term detrimental effect on mammalian cells, this treatment can become a useful tool for controlling microbial contamination in food processing and handling facilities without posing a risk to human health^22,23,48^. Unlike conventional UV and PL technologies, which are subject to strict regulatory limits for use in food applications ^49–51^, 405 nm blue light can be used at high doses without causing toxicity to humans operators. This makes 405 nm blue light a promising option for a continuous background treatment for microbial control in processing or storage areas, expanding the utilizations of germicidal light technologies.

**Table 1:** Weibull parameters for 405 nm LED inactivation kinetics of *E. coli*, *L. monocytogenes*, *S.* Typhimurium, *S. aureus* and *P. aeruginosa* suspended in thin liquid film of 1.2 mm thickness.

Substrate	Scale parameter *α*	Shape parameter *β*	*R*^2^
*E. coli*	0.09 ± 0.09 b	0.60 ± 0.39 A	0.77
*L. monocytogenes*	0.02 ± 0.02 a	1.02 ± 0.33 A	0.98
*S.* Typhimurium	0.06 ± 0.06 b	0.76 ± 0.35 A	0.94
*S. aureus*	0.05 ± 0.06 b	1.11 ± 0.48 A	0.99
*P. aeruginosa*	0.19 ± 0.12 b	0.76 ± 0.19 A	0.98

Values represent means ± 1 standard deviation (n = 3). Post-ANOVA pairwise comparisons (Tukey HSD) were conducted for Weibull model parameters *a* and *b* respectively. Disconnected letters indicate significant difference between the average values (*p* < 0.05).

Visible light inactivation has been credited to the photostimulation of endogenous intracellular porphyrins by light in the wavelength range 200 nm to 460 nm, with 400 nm to 420 nm being considered optimal for inactivation^52^. Stimulation of these porphyrins leads to the production of reactive species, predominantly singlet delta oxygen (^1^O2), a well-recognized trigger of cell death^53^. Nitzan et al.^54^ demonstrated that the predominant porphyrin produced in *S. aureus* and *Staphylococcus epidermidis* was coproporphyrin, whereas there was no predominant porphyrin produced in the gram-negative *E. coli*, *Acinetobacter*, and *Aeromonas* strains. The amount of coproporphyrin produced by staphylococcal strains was reported to be 2 to 3 times higher than in the gram-negative strains^54^. This agrees with the present results, which show a general trend of gram-positive bacteria requiring lower doses of 405 nm LED light for inactivation than gram-negative bacteria, which is in agreement with previous studies^39,40^. An exception was gram-negative *P. aeruginosa*, which was highly sensitive to the blue LED light, likely due to the production of coproporphyrin III and/or uroporphyrin III^55^. Among the gram-positive bacteria, the coproporphyrin content generated by *L. monocytogenes* was reported to be significantly lower than in *S. aureus*^56^, which can explain the lower inactivation rate of *L. monocytogenes* than *S. aureus* in TLF experiments. One possible explanation is that the amount of endogenous coproporphyrin generated in the bacterial cells varies not only between gram-positive and gram-negative bacteria, but also vary among bacteria within the same gram stain differentiation. Further studies are needed to elucidate the factors that influence the mechanism of endogenous porphyrin production and photodynamic inactivation of bacteria.

### Inactivation of bacteria on food contact surfaces and packaging materials

**Figure 2:**
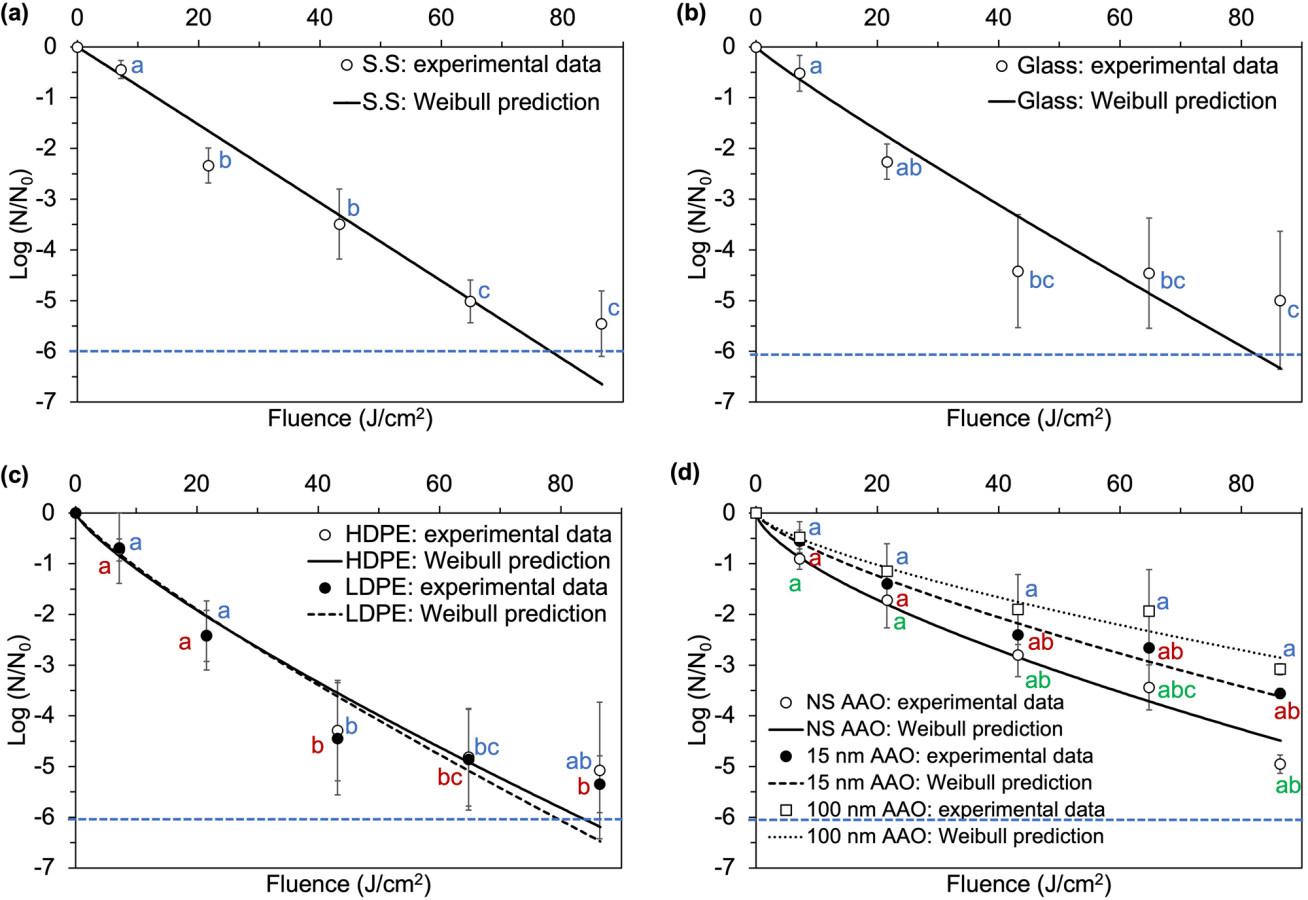
Experimental data and Weibull predicted inactivation curves for *E. coli* after exposure to continuous 405 nm LEDs of an irradiance of approximately 0.5 mW/cm^2^ on: (**a**) stainless steel (SS); (**b**) food-grade borosilicate glass (glass); (**c**) plastic materials: high density polyethylene (HDPE) and low density polyethylene (LDPE); and (**d**) anodic aluminum oxide with different nanotopography: nanosmooth (NS AAO), small nanopore (15 nm AAO) and large nanopore (100 nm AAO). Points represent means and error bars represent one standard deviation (n = 3). Limit of detection (LOD) is denoted by the blue dotted line. Points represent means and error bars represent one standard deviation (n = 3). Different letters denote significant differences (*p* < 0.05); inactivation data for the different colors were analyzed together at the same cumulative dose.

**Figure 3:**
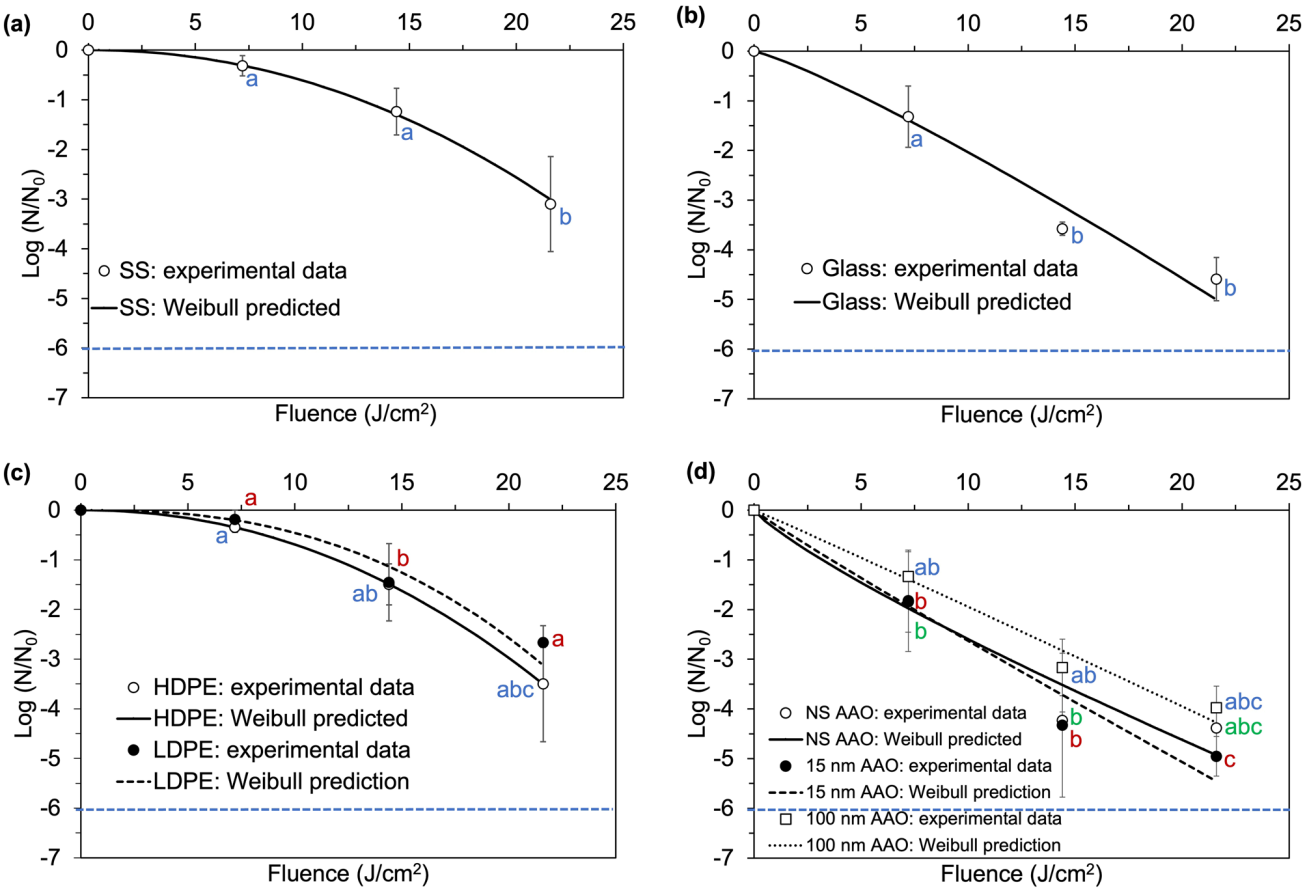
Experimental data and Weibull predicted inactivation curves for *L. monocytogenes* after exposure to continuous 405 nm LEDs of an irradiance of approximately 0.5 mW/cm^2^ on: (**a**) stainless steel (SS); (**b**) food-grade borosilicate glass (glass); (**c**) plastic materials: high density polyethylene (HDPE) and low density polyethylene (LDPE); and (**d**) anodic aluminum oxide with different nanotopography: nanosmooth (NS AAO), small nanopore (15 nm AAO) and large nanopore (100 nm AAO). Points represent means and error bars represent one standard deviation (n = 3). Limit of detection (LOD) is denoted by the blue dotted line. Points represent means and error bars represent one standard deviation (n = 3). Different letters denote significant differences (*p* < 0.05); inactivation data for the different colors were analyzed together at the same cumulative dose.

The gram-negative *E. coli* and the gram-positive *L. monocytogenes,* the more resistant species among the ones tested, were selected for 405 nm LED inactivation studies on solid substrates. The inactivation results are shown in Figs. 2 and 3. Significantly higher reduction of both *E. coli* and *L. monocytogenes* was obtained on all solid substrate materials, at each applied fluence level (*p* < 0.05), compared to the TLF experiments. This discrepancy may be due to differences in the experimental settings. Under the surface treatment conditions, uniform exposure of a small volume of bacterial inoculum was achieved, whereas for the static liquid treatments light may have been partially blocked by the edges of the rectangularly shaped chamber, shielding bacteria from the antimicrobial light. In a previous paper published by our group, Confocal microscopy images revealed that bacterial cells distribution varies significantly depending on the location within the suspending liquid^41^. When treating TLFs under static conditions, the bacterial cells on the bottom of the liquid suspension received a lower blue light dose compared to the cells at the surface of the suspension. Meanwhile, after the removal of the suspending liquid in case of the solid substrates, the bacterial population became homogenously distributed on the surface, allowing cells to have a more even exposure to the incident blue light. These two distinct spatial distributions of the bacterial cells inevitably result in very different light irradiance distribution within a liquid suspension *vs* a liquid-less bacterial pellet, which resulted in significant differences in inactivation in the two treatment scenarios, especially for *E. coli*.

For *E. coli* on SS, HDPE, LDPE, and glass surfaces, the reduction by blue LED was fast, resulting in an almost linear trend, with no visible plateau within the treatment period. In addition to these materials, which are typically used in food processing or clinical environments, a range of surfaces with controlled surface topography were also used. Previous studies have demonstrated that nanoporous surfaces with pore diameters of 15 and 25 nm can inhibit bacterial attachment^17,19^. In this study, we investigated inactivation of bacterial pathogens by blue LED light on surfaces with a range of nanoscale topographies. Maximum inactivation levels achieved on NS AAO, 15 nm AAO, and 100 nm AAO surfaces, were 5.0 ± 0.18, 3.6 ± 0.10, and 3.1 ± 0.11 log CFU, respectively.

For *E. coli* inactivation on AAO coupons experienced a fast initial inactivation within the first 24 h of exposure (43.2 J/cm^2^ cumulative dose), followed by a gradual plateau up to 48 h of exposure (86.4 J/cm^2^). When spread onto solid substrates, *L. monocytogenes* appeared to be more readily inactivated compared to *E. coli*, with near complete inactivation achieved after a cumulative dose greater than 21.6 J/cm^2^ (12 h). The bacterial counts were reduced below the limit of detection after exposure to 21.6 J/cm^2^ (12 h), on all substrates. No visible plateau was detected for the inactivation of *L. monocytogenes* on any solid substrate within duration of the treatment. Exposure to 405 nm resulted in 2.7 to 5.0 log reduction of *L. monocytogenes* on solid substrates, with the susceptibility decreasing in the following order: 15 nm AAO > Glass > NS AAO > 100 nm AAO > HDPE > SS > LDPE.

Since in practical applications it is useful to a priori predict microbial inactivation at any given light dose, the experimental inactivation data was used to generate quantitative Weibull kinetic parameters, for all inactivation scenarios. The calculated shape (*β*) and scale (*α*) parameters for the 405 nm LED treatments of *E. coli* and *L. monocytogenes* on different substrates are shown in Table 2. Due to the complexity of inactivation curves on solid substrates, different curvatures were observed for *E. coli* and *L. monocytogenes*. The value of *β* for *E. coli* on solid substrates was < 1 for all materials, with the exception SS. This reflects the high treatment susceptibility of *E. coli* on the SS surface. However, for inactivation of *L. monocytogenes* on solid substrates, concave down inactivation curves were observed (*β* > 1); an initial shoulder was present in the low dose region followed by faster reduction at higher doses. The shoulder indicates that cell death only started to occur after the accumulation of sublethal injury effects; once this surpassed a critical threshold, a rapid decline of survivors was observed. Because the bacterial suspension inoculated onto the solid substrates was dried before the treatment, the bacterial cells were exposed to the blue light treatment in a densely packed pellet, and the tailing effects were almost nonexistent. This is different than what was observed in previous studies conducted in liquid suspensions, in which inactivation curves of *E. coli*, *L. monocytogenes*, and *S. aureus* exhibited tailing, which was explained by the high turbidity and the aggregation of cells in the bacterial suspension^7,40,41,52^. The Weibull model was able to model accurately the inactivation curves for all substrate tested, and a good fit of the model to the experimental data was found for all three bacteria species (Tables 2, 3).

**Table 2:** Weibull parameters for 405 nm LED inactivation of *E. coli* and *L. monocytogenes* on different solid substrates.

Substrate	*E. coli*			*L. monocytogenes*		
	Scale parameter *α*	Shape parameter *β*	*R*^2^	Scale parameter *α*	Shape parameter *β*	*R*^2^
SS	0.08 ± 0.27 a	1.00 ± 0.31 A	0.95	0.01 ± 0.01 a	2.07 ± 0.60 A	1.00
HDPE	0.17 ± 0.16 a	0.80 ± 0.24 A	0.95	0.01 ± 0.01 a	2.11 ± 0.61 A	1.00
LDPE	0.15 ± 0.32 a	0.84 ± 0.59 A	0.95	0.00 ± 0.00 a	2.49 ± 1.31 A	0.98
Glass	0.10 ± 0.14 a	0.92 ± 0.48 A	0.93	0.14 ± 0.16 a	1.17 ± 0.60 A	0.97
NS AAO	0.24 ± 0.05 a	0.66 ± 0.06 A	0.99	0.38 ± 0.35 a	0.83 ± 0.43 A	0.89
15 nm AAO	014 ± 0.08 a	0.73 ± 0.16 A	0.99	0.30 ± 0.53 a	0.95 ± 0.40 A	0.94
100 nm AAO	0.12 ± 0.11 a	0.70 ± 0.21 A	0.97	0.19 ± 0.28 a	1.02 ± 0.41 A	0.97

Values represent means ± 1 standard deviation (n = 3). Post-ANOVA pairwise comparisons (Tukey HSD) were conducted for Weibull model parameters *a* and *b* respectively. Disconnected letters indicate significant difference between the average values (*p* < 0.05).

**Table 3:** Weibull parameters for 405 nm LED inactivation of *S. aureus* on anodic aluminum oxide (AAO) substrates.

Substrate	Scale parameter *α*	Shape parameter *β*	*R*^2^
NS AAO	0.06 ± 0.03 a	1.46 ± 0.22 A	0.99
15 nm AAO	0.10 ± 0.01 a	1.36 ± 0.06 A	0.94
100 nm AAO	0.16 ± 0.34 a	1.11 ± 0.52 A	0.98

Values represent means ± 1 standard deviation (n = 3). Post-ANOVA pairwise comparisons (Tukey HSD) were conducted for Weibull model parameters *a* and *b* respectively. Disconnected letters indicate significant difference between the average values (*p* < 0.05).

### Effect of physical properties of substrates on microbial inactivation

Some surface physical properties can influence the antimicrobial efficiency of light on solid substrates, adding an additional degree of unpredictability in the implementation of light disinfection systems. One goal of this study was to investigate and compare the microbial inactivation by 405 nm LED light on materials with different surface physical properties. Table 4 shows the surface roughness parameters, reflectivity, and water contact angles of the inert solid substrates used. Contact angles of all solid substrate surfaces were < 90°, indicating hydrophilic behavior, and decreased in the following order: HDPE (89.56°) > LDPE (87.63°) > 100 nm AAO (59.16°) > Glass (57.74°) > NS AAO (48.83°) > 15 nm AAO (44.58°) > SS (31.02°). Differences in water contact angles among some of these materials were statistically significant (*p* < 0.05) (Table 4). Reduction patterns of *E. coli*, *L. monocytogenes*, and *S. aureus* showed a strong correlation with the contact angles of the solid substrates: the more hydrophilic the surface, the higher the log reduction. The HDPE and LDPE surfaces had contact angles > 65°. The hydrophobic character of these surfaces led to aggregation of bacterial cells in the liquid that beaded on the surfaces, which resulted in dense, stacked layers of bacteria that provided a pronounced shading effect^9^. The inoculum on the hydrophilic SS surface spread over a larger area compared to the hydrophobic surfaces, and thus after the liquid removal bacteria cells were distributed on the surface in thinner layers, which facilitated easier penetration of light and a more uniform light exposure. This is further corroborated by the fact that the highest overall inactivation of *E. coli* and *L. monocytogenes* was observed on the most hydrophilic surfaces (SS and 15 nm AAO), while the lowest inactivation was obtained on the more hydrophobic surfaces (100 nm AAO and LDPE). The highest overall inactivation of *S. aureus* on solid substrates was found on the 15 nm AAO surface, the most hydrophilic among the AAO surfaces.

**Table 4:** Measured surface physical properties of the solid substrates: surface roughness parameters, surface reflectivity percentages, water contact angle.

Solid Substrates	Surface roughness parameters (µm)		Surface reflectivity (%)		Water contact angle (°)
	S_a_	S_q_	Diffuse	Specular	
SS	1.04 ± 0.09 b	11.27 ± 0.50 ab	59.48 ± 0.57 cd	32.20 ± 1.14 c	31.02 ± 6.59 d
HDPE	1.31 ± 0.07 a	13.59 ± 1.04 a	59.98 ± 0.10 c	6.91 ± 0.34 e	89.56 ± 2.93 a
LDPE	0.21 ± 0.15 e	4.72 ± 0.92 c	61.12 ± 0.05 b	10.49 ± 0.09 d	87.63 ± 4.34 a
Glass	0.02 ± 0.00 f.	0.46 ± 0.09 d	45.19 ± 0.09 e	11.03 ± 0.19 d	57.74 ± 3.88 b
NS AAO	0.60 ± 0.06 d	8.78 ± 1.78 b	67.82 ± 0.14 a	31.86 ± 0.16 c	48.83 ± 4.00 bc
15 nm AAO	0.84 ± 0.08 c	9.61 ± 1.74 b	59.01 ± 0.06 d	40.46 ± 0.08 b	44.58 ± 3.83 c
100 nm AAO	0.76 ± 0.06 cd	9.91 ± 0.55 b	44.45 ± 0.05 f	49.17 ± 0.04 a	59.16 ± 3.02 b

Values represent means ± 1 standard deviation (n = 3). Post-ANOVA pairwise comparisons (Tukey HSD) were conducted for different surface physical properties. Disconnected letters indicate significant difference between the average values (*p* < 0.05).

Roughness affects the cleanability and hygienic status of surfaces. Surfaces with lower roughness tend to be more hygienic, since they are less likely to harbor residues and microorganisms^57,58^. In this study, no strong correlation between surface roughness parameters and inactivation by LED light was observed. S_a_ and S_q_ of HDPE showed the highest values (1.31 and 13.59 µm), followed by SS (1.04 and 11.27 µm), 15 nm AAO (0.84 and 9.61 µm), 100 nm AAO (0.76 and 9.91 µm), NS AAO (0.60 and 8.78 µm), LDPE (0.21 and 4.72 µm), and glass (0.02 and 0.46 µm). The roughness parameters differed significantly among materials (*p* < 0.05). The highest inactivation of *E. coli* and *L. monocytogenes* was observed on SS and small pore AAO (15 nm) surfaces, although the smallest surface roughness parameters were measured on glass surface. While S_a_ and S_q_ values have some practical usefulness, these values do not differentiate between peaks and crevices on a surface^59^. Surface imperfections such as crevices and valleys have higher relevance for inactivation of bacteria by light because they are able to shield and protect the cells from the light exposure. Previous studies have also shown that more retention and stronger adhesion of bacteria occurs for surfaces with in crevices and imperfections of sizes comparable to bacteria sizes compared to flat surfaces or surfaces with rougher features, due to the high contact area between the cells and the substrate^22,60^. Park & Kang presented scanning electron microscopy images showing pathogenic bacterial cells aggregated in cracks and crevices of comparable size on food contact surfaces, which could protect these cells against decontamination measures^61^.

Another surface physical property that could impact the effectiveness of light treatments is surface reflectivity. Table 4 shows the specular reflectivity (light reflected at the incident angle) and diffuse reflectivity (light reflected as a different angle than the incident angle) values of the surfaces at a spectral wavelength of 405 nm. The diffuse reflectivity decreased in the following order: NS AAO (67.82%) > LDPE (61.12%) > HDPE (59.98%) > SS (59.48%) > 15 nm AAO (59.01) > Glass (45.19%) > 100 nm AAO (44.45%). Specular reflectivity of 100 nm AAO showed the highest values (49.17%), followed by 15 nm AAO (40.46%), SS (32.20%), NS AAO (31.86%), Glass (11.03%), LDPE (10.49%), and HDPE (6.91%). There were statistically significant differences among the tested substrate materials both in terms of diffuse and specular reflectivity (*p* < 0.05). The highest specula^r^ reflectivity was observed for 100 nm AAO and 15 nm AAO surfaces, while NS AAO surface had the highest diffuse reflectivity. These three surfaces have the same material composition, but they differ in surface topography. There was just a weak correlation between surface reflectivity and inactivation of *L. monocytogenes* and *S. aureus*. However, the lowest overall inactivation of *E. coli* was achieved on the highly reflective AAO surfaces, which agrees with a previously reported negative correlation between surface reflectivity and microbial inactivation^62,63^.

The overall effects of solid substrate physical properties on inactivation by the blue light LED treatment were also analyzed using a stepwise multiple regression model, with the cumulative log reduction as the dependent variable and the surface physical property parameters as independent variables (Table 5). Based on the values of the cumulative coefficient of determination for the multiple regression model *R*^*2*^, the measured surface physical property parameters impacted the inactivation by 405 nm LED light in the following decreasing order: water contact angle > surface reflectivity > surface roughness for *L. monocytogenes* and *S. aureus*, and surface reflectivity > water contact angle > surface roughness for *E. coli*. No correlation of inactivation with any interaction terms was found. This indicates that the contact angle and surface reflectivity percentages are better predictors of the cumulative inactivation, while surface roughness had the lowest correlation with the inactivation of *E. coli*, *L. monocytogenes*, and *S. aureus*.

**Table 5:** Stepwise multiple regression of the effects of measured surface physical property parameters on the cumulative inactivation by 405 nm LED treatments for *E. coli*, *L. monocytogenes*, and *S. aureus*.

Model: Log reduction = *f* [surface roughness (S_a_, S_q_), water contact angle, surface reflectivity % (diffusive, specular)]			
Variable added to regression	Multiple *R*^2^		
	*E. coli*	*L. monocytogenes*	*S. aureus*
S_a_	0.014	0.001	0.233
S_q_	0.079	0.032	0.250
Water contact angle	0.082	0.126	0.943
Diffuse reflectivity	0.295	0.152	0.944
Specular reflectivity	0.558	0.158	0.949

### Inactivation of bacteria on substrates with controlled surfaces nanotopography

Surface modification is an emerging strategy for preventing bacteria attachment and biofilm growth on abiotic surfaces. Anodic alumina (AAO) surfaces with nanopores of 15–25 nm diameters has shown to significantly reduce biofouling by various foodborne pathogens^42,64^, rendering these AAO surfaces useful in food safety, biomedical, and water treatment applications^65,66^.

**Figure 4:**
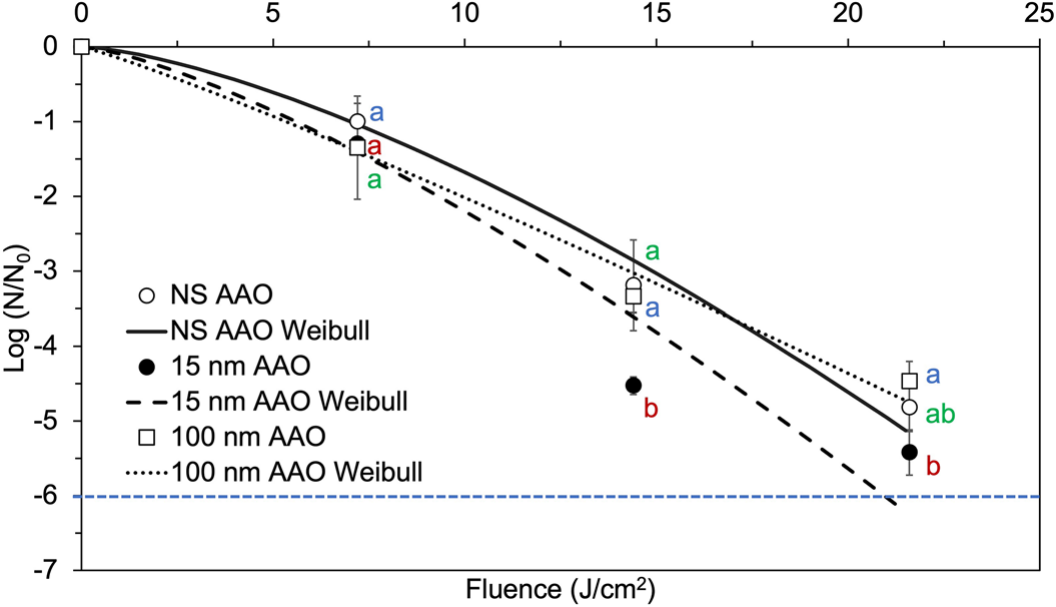
Experimental data and Weibull predicted inactivation curves for *S. aureus* after exposure to continuous 405 nm LEDs of an irradiance of approximately 0.5 mW/cm^2^ on anodic aluminum oxide substrates with different nanotopography: nanosmooth (NS AAO); small nanopore (15 nm AAO) and large nanopore (100 nm AAO). Data points represent means and error bars represent one standard deviation (n = 3). Limit of detection (LOD) is denoted by the blue dotted line. Different letters denote significant differences (*p* < 0.05); inactivation data for the different colors were analyzed together at the same cumulative dose.

The results of this study demonstrate that the nanoporous topography had an enhancing effect on the inactivation *L. monocytogenes* and *S. aureus* by 405 nm LED treatment (Figs. 3, 4). *S. aureus* counts were reduced to below the limit of detection (100 CFU/coupon) after a cumulative fluence of 21.6 J/cm^2^ (12 h exposure), on all AAO substrates (Fig. 4). *S. aureus* experienced a slow reduction by the 405 nm LED treatment up to a 7.2 J/cm^2^ cumulative dose, followed by a fast reduction, for all AAO surfaces. A 12 h exposure (21.6 J/cm^2^ cumulative dose) resulted in 4.5- to 5.4-log reduction on AAO surfaces with different pore sizes. The inactivation data was fitted using the Weibull model, and an excellent fit of the data with the model was obtained, with 0.94 ≤ *R*^2^ ≤ 0.99 (Table 3). No inactivation plateau was observed for treatments of up to 25 J/cm^2^ cumulative fluence, which suggests that the maximum inactivation possible has not been reached within the treatment dose used in this work. The inactivation curve of *S. aureus* showed a concave down trend, and a Weibull shape parameter *β* > 1 for all tested AAO surfaces (Table 3). The fast reduction of *S. aureus* in TLF and on solid AAO surfaces indicates the high susceptibility of *S. aureus* to 405 nm LED light.

Another observation is that inactivation on small nanopore (15 nm) AAO surfaces was significantly higher compared to nanosmooth (NS AAO) and large nanopore (100 nm AAO) surfaces, at all light doses. The higher inactivation on small nanopore AAOs may be due to the hydrophilic character of these surfaces, which promoted the spreading of the bacterial inoculum. By contrast, the liquid inoculum beaded up on the more hydrophobic 100 nm pore surfaces, leading to a pronounced clustering of bacterial cells, which shielded them from the blue light. The intrinsic antifouling effects of AAO surfaces, combined with their inactivation enhancing effect during 405 nm LED treatments, makes them well-suited for a variety of applications where biofouling and microbial contaminations are major concerns, including food, biotechnology, and healthcare industries.

## Conclusions

This study demonstrates the potential of 405 nm blue LEDs as an effective decontamination treatment for standing liquids, food contact surfaces, and packaging materials in food processing or handling environments, as well as a range of surfaces in clinical settings. The non-toxicity of 405 nm light, its ability to work under refrigeration conditions, and the flexible design afforded by LED systems open numerous opportunities for this technology as an alternative to UV systems or other disinfection solutions. The additional beneficial impact of small size surface nanotopography can be used to design hurdle systems consisting of a combination of antifouling surfaces and 405 nm light disinfection can be used to develop novel antimicrobial applications in the food and healthcare industries.

## Materials and methods

### Bacterial cultures

The bacterial strains used in this study were *L. monocytogenes* serotype 1/2a strain 10403s (one of the most prevalent strain in foods and food processing environment^67,68^), *E. coli* serotype O157:H7 ATCC43895 (a ground beef isolate from 1983 hemorrhagic colitis outbreak in Michigan^69,70^), *S.* Typhimurium FSL S90123 (an environmental isolate kindly provided by the Food Safety Laboratory at Cornell University (Ithaca, NY, USA)^60^), *S. aureus* ATCC9144 (one of the major prevalent foodborne *S. aureus* strains^71^), and *P. aeruginosa* ATCC15442 (an environmental strain isolated from animal room water bottle^72,73^), obtained from American Type Culture Collection (Manassas, VA). Prior to the experiments, all cultures were streaked onto tryptic soy agar (TSA) from frozen stock (−80 ℃) and incubated for 24 h at 37 ℃. A single isolated colony was then transferred into 3 mL of tryptic soy broth (TSB) for passage one (37 ℃, 24 h). Thirty µL of grown passage one culture was transferred to fresh 3 mL TSB for passage two (37 ℃, 18 h). Bacteria suspension in stationary phase was centrifuged at 5000 RPM (1957×*g*) for 10 min at 21 ℃, and the pellet was resuspended in Butterfield Phosphate Buffer (BPB, pH 7.2) for three times total to ensure minimal remnants of TSB in the final bacteria suspension. The initial inoculum level was about 10^9^ CFU/mL for all strains.

### 405 nm LED treatment apparatus

**Figure 5:**
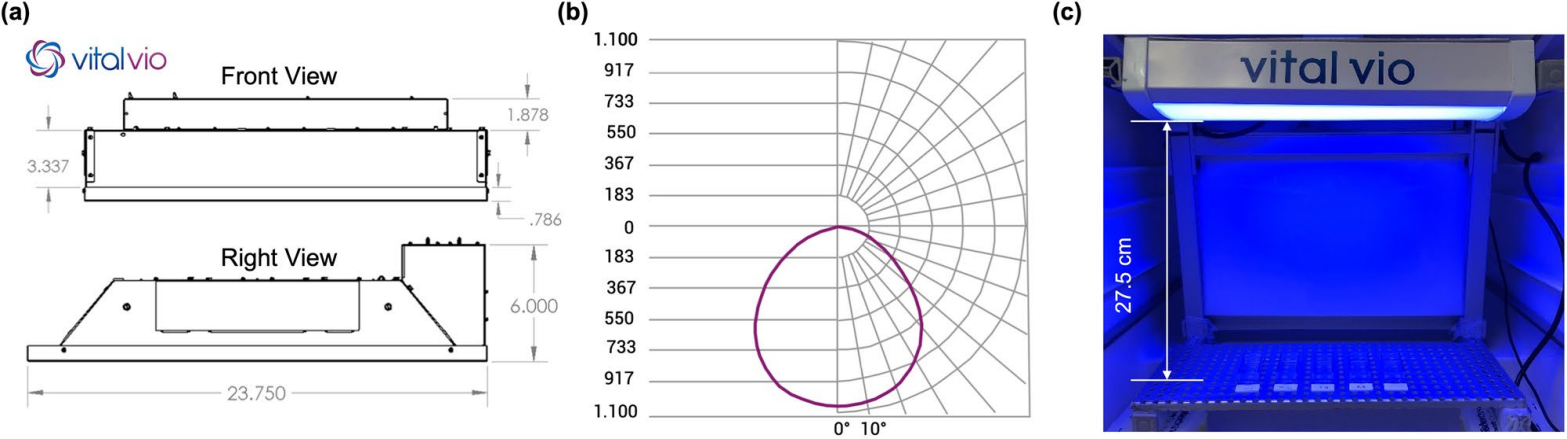
The 405 nm blue LED experimental set up. (**a**) Dimensions of the 405 nm blue LED panel. (**b**) Light intensity emission (in Candela) tested at the lamp surface, at 25 °C. (**c**) Lamp setup used in the bacterial inactivation experiments.

All inactivation experiments were performed using a Vital Vio VVLD22^®^ LED unit (Vital Vio, Troy, NY). This apparatus has a rectangular LED array that delivers monochromatic light with an output emission spectrum centered at 405 nm (full width at half maximum = 14 nm). The LED rig was kept in a 4 ± 2 ℃ temperature-controlled incubator at all times, to prevent any heating of the tested samples due to exposure to the LED source, and to mimic inactivation under refrigeration conditions, which is highly relevant for food applications. A digital thermometer was used to record the environmental temperatures throughout the 405 nm LED treatments. The rectangular LED array (60.3 cm × 8.48 cm) was set in a fixed position, at 27.5 cm directly above the target surfaces, to provide a good balance between intensity of irradiance and homogeneity of the light distribution (Fig. 5). The LED unit was powered by a DC power supply (120–277 V), giving an approximate irradiance of 0.5 mW/cm^2^ at the targeted surface. Several treatment durations were chosen to deliver different dosage of 405 nm light: 4 h, 8 h, 12 h, 24 h, 36 h, 48 h, corresponding to cumulative fluences of 7.2 J/cm^2^, 14.4 J/cm^2^, 21.6 J/cm^2^, 43.2 J/cm^2^, 64.8 J/cm^2^, and 86.4 J/cm^2^, respectively. In the bacterial inactivation experiments, different samples from the same bacterial culture were prepared for each treatment duration, hence the corresponding sample can be taken out at specific time point to be enumerated and counted.

### Bacterial inactivation by 405 nm LED treatments in different substrates

#### Treatment of thin liquid films

To mimic contaminated standing water in food processing or food service environments, 1 mL of bacteria suspension was transferred into Nunc Lab-Tek™ II 1 well Chamber Slides™ (17 mm × 48 mm, Fisher Scientific, Rochester, NY), in the form of a thin liquid film. Prior to use, these chambers were soaked in 70% ethyl alcohol for 24 h for decontamination, followed by 2 h of drying in a biosafety cabinet to evaporate the remaining ethyl alcohol. The bacterial suspensions were allowed to equilibrate for 3 min prior to the 405 nm light exposure. To reduce the effect of excess drying on inactivation efficiency, during the light treatments all chambers were sealed with low density polyethylene (LDPE) (Uline, Waukegan, IL), which is highly transmissible for blue light. The inactivation kinetics for the 405 nm LED treatment on *E. coli*, *L. monocytogenes*, *S. aureus*, *P. aeruginosa*, and *S.* Typhimurium was investigated by exposing 1 mL of bacteria containing liquid film (thickness = 1.2 mm) for the durations specified previously, except the 8 h duration, which was only used for the solid surface experiments. The survivors from both 405 nm light treated, and untreated control samples were determined using the standard plate counting method on TSA agar. Plates were incubated at 37 ℃ for 24 h, after which the survivors were enumerated, and results reported as colony forming units (CFU/mL). Log reduction was calculated using the following equation:

1 $$Log\, Reduction=Log(N/{N}_{0})$$

where *N*_0_ and *N* are bacterial counts (in CFU per mL of suspension) before and after 405 nm light treatment, respectively. The detection limit for all strains in TLF experiments was 100 CFU per spread agar plate.

### Treatment of solid food contact surfaces, packaging materials, and nanoporous anodic aluminum oxide surfaces

Rectangular (10 mm × 25 mm) coupons of the following materials were used: high density polyethylene (HDPE) (6.45 mm thickness; Regal Plastics, Dallas, TX), LDPE (0.14 mm thickness, Uline, Waukegan, IL), food grade borosilicate glass (0.96 mm thickness, Fisher Scientific, Rochester, NY), and food-grade stainless steel with a glass bead blast finish (SS) (1.45 mm thickness, Fountain Valley, CA). Prior to inoculation, all coupons were sequentially sonicated (40 kHz, Branson 1210 Ultrasonic Cleaner, Branson Ultrasonics, Danbury, CT) in 95% acetone (Fisher Scientific, Rochester, NY), 95% ethyl alcohol, and deionized water, for 15 min at each step, to remove any residues and inactivate any potential microbial surface contaminants. The cleaned and sanitized coupons were then rinsed with sterile deionized water and dried at room temperature in a biosafety cabinet. A total of 100 µL bacteria suspension was aliquoted as one spot inoculation onto the coupon surfaces. The inoculated coupons were placed in sterile polystyrene Petri dishes (Fisher brand, Pittsburgh, PA) and left in a laminar flow hood (23 ℃, 17%RH) for 3 h to dry, until they reached a constant weight^41^. During the 405 nm light treatment, all coupons were covered with 405 nm light transmitting Low Density Polyethylene of 0.14 mm thickness (Uline, Waukegan, IL) to prevent excessive drying.

To recover the bacteria from the treated surfaces, all treated coupons were individually placed in sterile WhirlPak bags with 10 mL BPB and sonicated for 5 min at 40 kHz (Branson 1210 Ultrasonic Cleaner, Branson Ultrasonics, Danbury, CT). It has been shown before that this step has minimal effect on bacterial viability^74^. It is important to note that the cell recovery method used in the present study has been reported before and was proven to result similar recovery losses with no statistical differences (*p* > 0.05) among materials^41,75^. This is important to note, as it indicates that any differences in inactivation among substrates cannot be attributed to varying recovery losses of cells from these different substrates. The same recovery procedure was also used for the control groups. Samples were then taken from the resulting BPB, and survivors quantified by standard plate counting, as described above. The inactivation results on solid substrates described in this study were reported as colony forming units per area (10 mm × 25 mm) of each coupon used, as abbreviated as CFU/coupon in the following sections. The detection limit for bacterial counts for the surface treatments was 100 CFU per coupon for all strains. Microbial reduction results were calculated using Eq. (1). Technical duplicates were performed for each type of coupon; all LED treatments were performed in triplicate, with independently grown bacterial cultures.

In addition to the common food contact surfaces and packaging materials mentioned above, we tested the effects of 405 nm blue LED treatments on contact surface materials relevant to clinical and healthcare applications. Nanoporous anodic aluminum oxide (AAO) surfaces with pore diameters of 15 and 100 nm were prepared by two-step anodization of high purity aluminum (99.99%, Alfa Aesar, Ward Hill, MA), as described before^42^. Briefly, the Aluminum substratum was first subjected to mechanical polishing, annealing, and electrical polishing. Both anodization steps were carried out in 0.3 M oxalic acid (Beantown Chemical, Hudson, NH) with stirring at (75 RPM) at 16 °C, which was maintained by a circulating water bath. The first porous aluminum oxide layer was etched away, then the second anodization procedure was performed, during which pore growth was initiated from dents left over by the nanopores in the first layer, resulting in regular surface features. Pore size was controlled by voltage and post-anodization pore widening procedure in H_3_PO_4_ (0.1 M, 30 ℃, 70 RPM). For 15 nm diameter nanopores, 10 V voltage was applied in both anodization steps and no pore widening procedure was performed due to the small pore diameters targeted. For 100 nm diameter nanopores, 60 V and 50 V voltages were applied to the first and second anodization steps, respectively, and a subsequent pore widening procedure was performed (0.1 M H_3_PO_4_, 30 ℃, 70 RPM, 40 V)^42, 64^. Nanosmooth aluminum oxide surfaces of 1 × 2.5 × 0.5 mm (Alfa Aesar, Haverhill, MA) were used as control without any surface nanopores.

### Modelling of inactivation kinetics

The kinetics of microbial inactivation by the 405 nm LED treatments was described using the Weibull model^76^:

2 $$Log(N/{N}_{0})=\alpha {t}^{\beta }$$

where $N/{N}_{0}$ represents the ratio of survivors after treatment over the initial population,* α* is the scale parameter, which describes the magnitude of log_10_ change, and *β* is the shape factor, which describes the shape of the inactivation curves. A shape parameter *β* > 1 describes a concave down curve, *β* < 1 describes a concave up curve, and *β* = 1 describes a linear inactivation curve. The *α* and *β* were evaluated from the intercept (i.e., log(*α*)) and slope (i.e., *β*) of the linear regression of the linearized dataset (i.e., $\mathrm{log}[\mathrm{log}\left(\frac{N}{{N}_{0}}\right)]$ vs. $\mathrm{log}(\mathrm{t})$). Parameter fitting was conducted by linear regression using Minitab software release 19.

### Surface property analyses of the solid substrates

Surface hydrophobicity was assessed by measuring water contact angles using a Ramé-Hart 500 Advanced Goniometer/Tensiometer (Ramé-Hart Inc., Succasunna, NJ) with reagent grade deionized water at room temperature on cleaned and sterilized coupons, as described before^77^. The data was analyzed using the instrument’s DROPimage software. All measurements were performed in triplicate and average values of contact angles were used as a measure of surface hydrophobicity. Contact angle values smaller than 90° indicate a hydrophilic surface, and values larger than 90° indicate a hydrophobic surface^8^. This measurement of hydrophobicity was used to evaluate the tendency for surface spreading of the water based liquid inoculum.

Surface roughness of all substrates was measured using a Keyence VK-X260 Laser-Scanning profilometer, at the Cornell Center for Materials Research (Ithaca, NY). The following roughness parameters were determined: S_a_, the extension of R_a_ (arithmetical mean height of a line) to a surface, which expresses the average roughness, and represents the difference in height of each point compared to the arithmetical mean; and S_q_, the sum of the largest peak height value and the largest pit depth value within the defined area. Measurements were conducted on a 5 mm length of the sample, which was scanned with an applied stylus force of 4.47 mg. Triplicate measurements were performed for each material.

Specular and diffuse reflection profiles of the clean coupons were measured using a Cary 5000 UV–Vis-NIR spectrophotometer with the integrating sphere diffuse reflectance accessories (Agilent, Santa Clara, CA). The data was analyzed using the instrument’s Cary WinUV software. Reflectance measurements were made by mounting the coupons on the integrating sphere wall, ensuring efficient collection of a high proportion of reflected radiation. Specular reflection was measured by having the detector at 90° from coupon surfaces, while diffuse reflection measurements were conducted when detector was at 45° relative to the coupon surfaces. All measurements were performed in triplicate.

### Statistical analysis

Mean values of data were obtained from three independent trials, each with technical duplicates (6 values for each data point). Analyses of variance and post hoc Tukey’s HSD were used evaluate differences in log reduction, Weibull kinetic parameters, and physical properties among materials and different treatment levels. A confidence level of 95% was adopted for all statistical tests. Cluster analysis was performed on the inactivation data obtained from the TLF experiments to determine the similarity in inactivation levels reached by various strains. Multivariate analysis was performed using a stepwise regression model to determine the independent effect of each measured surface physical property on the cumulative inactivation results. All statistical analyses were performed using Minitab software release 19.

## Data Availability

The datasets generated during and/or analyzed during the current study are available from the corresponding author on reasonable request.
